# Interaction of perforin and granzyme B and HTLV-1 viral factors is associated with Adult T cell Leukemia development

**DOI:** 10.22038/ijbms.2020.38454.9602

**Published:** 2020-08

**Authors:** Mohammad Mehdi Akbarin, Sadegh Farhadi, Abolghasem Allahyari, Mohammad Mehdi Koshayar, Abbas Shirdel, Hossein Rahimi, Seyed Abdolrahim Rezaee, Maryam Mahdifar, Zahra Mozaheb, Asadollah Mohamadi, Alireza Bari, SeyedehTahereh Mohaddes, Houshang Rafatpanah

**Affiliations:** 1 Immunology Research Center, Inflammation and Inflammatory Diseases Division, Mashhad University of Medical Sciences, Mashhad, Iran; 2 Hematology Department, Ghaem Hospital, Faculty of Medicine, Mashhad University of Medical Sciences, Mashhad, Iran; 3 Hematology Department, Imam Reza Hospital, Faculty of Medicine, Mashhad University of Medical Sciences, Mashhad, Iran

**Keywords:** ATL, Granzyme, HTLV-1, Perforin, Proviral load

## Abstract

**Objective(s):**

Human T cell leukaemia virus type 1 (HTLV-1) is associated with adult T cell leukaemia (ATL), a malignant lymphoproliferative disease that infects CD4 T cells. It is not clear why the majority of HTLV-1-infected individuals remain asymptomatic carries (ACs) and a minority develop ATL. Cellular immune response has a critical role in ATL and destroys malignant and HTLV-1-infected cells. Perforin and granzyme have important functional roles in apoptosis and destruction of infected cells. In the present study we examined the role of perforin and granzyme in ATL patients and ACs.

**Materials and Methods:**

Peripheral blood mononuclear cells (PBMCs) were isolated from ATL patients and ACs by using Ficoll-hypaque density centrifugation. RNA was extracted and cDNA was synthesized. A real-time PCR TaqMan method was designed and optimized for evaluation of perforin, granzyme, tax, and HBZ gene expression. HTLV-1 proviral load (PVL) was quantified in patients with ATL and ACs.

**Results:**

The mRNA expression of tax and HBZ was significantly higher in ATL patients than ACs (*P*=0.011 and *P*=0.0001,respectively). The HTLV-1 PVL was higher in ATL patients compared to with AC group (*P*=0.015). There was a significant increase in perforin gene expression in ACs compared with ATL patients (*P*=0.002). Furthermore, the expression of granzyme was also higher in ACs compared with ATL patients, and significant differences were observed between the two groups (*P*=0.036).

**Conclusion:**

Low expression of perforin and granzyme in ATL patients seems to influence the efficiency of CTL function and destruction of HTLV-1-infected cells, which might contribute to the disease pathogenesis.

## Introduction

Human T cell leukemia virus type 1 (HTLV-1) belongs to the Retroviridae family which is associated with two main types of diseases known as HTLV-1-associated myelopathy/tropical spastic paraparesis (HAM/TSP) and Adult T cell Leukemia (ATL) (1). It is estimated that around 10–20 million people are infected worldwide with HTLV-1, with the virus being more prominent in some areas such as Japan, the Caribbean, Northeast of Iran, Central Africa, and several regions of South America (2, 3). Cities of Mashhad, Nayshabur, and Sabzevar in the Northeast of Iran have been recognized as endemic regions for HTLV-1 infection (2, 4). 

ATL is a poor prognosis peripheral T cell malignancy affecting various organs such as the skin, lungs, liver, spleen, and lymphoid glands (5, 6). Most of the malignant T cells express CD2, CD3, CD4, CD5, CD25, and HLA-DR (7). Tax and HBZ are the main viral transactivating elements, having a critical role in leukemogenesis and cell growth disruptions (8). Previous studies have demonstrated that Tax and HBZ induce cell signaling pathways via, AKt and NF-κB. Furthermore, these viral molecules are known to disrupt the apoptosis process, cell cycle checkpoints, and DNA repair molecules including retinoblastoma and P53 (9). 

Strong humoral and cellular immune responses are induced by Tax and other HTLV-1 products which determine the outcome of HTLV-1 infection in the infected individuals (10, 11). It has been well known that both natural killer cells (NKCs) and cytotoxic T lymphocytes (CTLs) kill HTLV-1-infected cells by cell-mediated cytolysis mechanism (12). Perforin and granzyme are two major components of cell-mediated immunity which has pivotal roles in the elimination of virus-infected cells (13). 

Perforin is a cytolytic killer cell mediator, released from the cytolytic granules which are structurally similar to the complement component 9 (14). Granzyme B, belonging to serine protease proteins, is associated with perforin as a delivery element and induces apoptosis in the target cell. This protein can degrade nucleic acids by direct attachment and cleavage of executive caspases 3 (14, 15). Furthermore, granzyme B blocks anti-apoptotic pathways by disrupting Bid and Bax proteins which leads to the release of the cytochrome c from the mitochondria membrane, resulting in apoptosis acceleration (15). 

Compared with ATL patients, CTL response is more efficient in ACs as it is able to prevent viral replication and spread of the virus (16). It has also been shown that perforin and granzyme B expression in HTLV-1-specific CD8^+^ T cells of ATL patients is significantly lower than that of ACs suggesting that decreased frequency, diversity, and function of HTLV-1 specific CD8+ T cell clones are associated with the risks of ATL development (17). Furthermore, the frequency of HTLV-1-specific CD8^+^ T cells with poor lytic capacity is higher in HAM/TSP patients, whilst healthy ACs exhibit lower frequencies of cells with high lytic capacity (18). The present study was conducted to determine the correlation between perforin and granzyme and viral factors including Tax, HBZ, and proviral load (PVL). 

## Materials and Methods

***Study population***

The study population included 19 ATL patients (11 females and 8 males) and 13 HTLV-1 ACs (8 females and 5 males) referred to the Hematology-Oncology department of Imam Reza and Ghaem Hospitals, Mashhad University of Medical Sciences, Mashhad, Iran, between January 2012 and December 2014. The demographic data and clinical features of patients such as organomegaly, lymphadenopathy, and coetaneous lesions were collected. The study was approved by the ethics committee of Mashhad University of Medical Sciences and consent form was taken from each participant (grant no. 920617). Moreover, WBC count and the presence of reactive lymphocytes were examined in ATL subjects. EDTA whole-blood samples were collected before any treatment proceedings and chemotherapy.

***PBMCs isolation and RNA extraction***

Peripheral blood mononuclear cells (PBMCs) were isolated using a Ficoll density gradient (Inno-train, Germany). Total RNA was extracted from PBMCs using a Tripure isolation kit (Roche, Germany) according to the manufacturer’s instructions and purified RNA was treated with DNase I to remove any genomic DNA contamination. RNA integrity was confirmed using gel electrophoresis.

***cDNA synthesis***

Complementary DNA (cDNA) was synthesized using random primers and reverse transcriptase according to the manufacturer’s instructions (Bioneer, South Korea). The reaction condition was repeated for 12 cycles as follows: 30 sec at 24 ^°^C, 4 min at 44 ^°^C, and 30 sec at 55 ^°^C. GAPDH primers were used to check for the correct cDNA synthesis. The GAPDH primers were as follows; forward: 5’-CAAGGTCATCCATGACAACTTTG-3’ and Reverse GAPDH primer 5’-GTCCACCACCCTGTTGCTGTAG-3’.

***Primer designing***

Primer and probe were designed for tax, HBZ, perforin, and granzyme B based on sequence data available on NCBI databases using Allele ID (version 5) software. Table 1 shows the specific primers and probes.

***Real time PCR***

Real-time PCR (Taqman method) was performed on a Rotor gene 6000 cycler (Corbett, Hilden, Germany). All samples were performed in duplicate and related to the expression of an appropriate housekeeping gene, β2 Microglobulin. The PCR reaction consisted of primary heating at 95 ^°^C for 4 min, followed by 45 cycles of denaturation (15 sec at 94 ^°^C) and annealing 20 sec at optimum temperature and extension phase 20 sec at 72 ^°^C.

***Proviral load measurement***

PBMCs were isolated by Ficoll density gradient (Cedarlane Laboratories, Hornby, Canada). To assess HTLV-1 PVL, DNA was extracted from PBMCs using an available commercial kit (Qiagen, Germany) and quantitative Real-time PCR was carried out using a commercial absolute quantification kit according to the manufacturers’ instructions as previously described (Novin Gene, Iran) (19). The number of HTLV-I proviruses was calculated and reported per 10^4^ PBMCs.

***Statistical analysis***

Data analysis was performed using SPSS (version 16). The results are presented as mean±SD. Kolmogorov-Smirnov test was used to examine normality of distribution. Statistical analyses were performed in two groups using the Mann-Whitney U test. Spearman’s rank correlation tests were used for the relation between parameters, *P-*values less than 0.05 were considered to be statistically significant.

## Results

The mean ages in ATL patients and ACs were 53(46.87–69.93) and 39(22.7–49.93) years, respectively. According to clinical and physical examinations, most of the patients were considered to be in the acute phase. 61% of ATL patients showed lymphadenopathy, 21% suffered from opportunistic infections such as candidiasis, and 18% had skin lesions.

***High viral factors in ATL patients compared with ACs***

 The mean tax/β2 gene expression was higher in ATL subjects than in those of the ACs group and a significant difference was observed between the two groups (*P*=0.011) (Table 2). The mean of HBZ/β2 gene expression was significantly higher (1924-fold change) in ATL patients compared with ACs (*P*=0<0.1) (Table 2). The mean HTLV-1 PVL in ATL patients was 12525.61-±4121.24 copies/10^4^ and the percentage of HTLV-1 infected PBMCs in the ATL group was 125±41. In ACs, the mean HTLV-1 PVL was 454.30-±189.942 copies/10^4^ and the percentage of infected PBMCs was 4.54±1.89. Significant differences in PVL were observed between the two groups (*P*=0.015) (Table 3).

***PBMCs of ATL patients are associated with high expression of perforin and factors gene expression in ACs***

The mean perforin/β2 gene expression was significantly decreased in ATL patients compared with ACs (*P*=0.002) (Table 4). Granzyme/β2 gene expression mean value in ATL patients was significantly lower than that of the ACs group (*P*=0.036) (Table 4). 

***Gene expression correlation***

Positive correlation between tax and HBZ genes expression was observed (*P*=0.026, r= 0.571). Perforin and granzyme B gene expression had a direct affirmative correlation (*P*=0.005, r=0.587). No statistically significant correlations were found between HTLV-1 PVL, tax and HBZ gene expressions *(P=*0.9 and 0.12). Furthermore, perforin and granzyme B gene expressions were not affected by PVL, tax, and HBZ gene expressions (*P*>0.05).

**Table 1:** Primers and probes of tax, HBZ, perforin, granzyme, and β2 microglobulin genes

Gene	Forward primer( 5′-3′)	Reveres primer ( 5′-3′)	Probe ( 5′-3′) Fam-BHQ1
tax	CCCTAATAATTCTACCCGAGGACTG	GCCATCGGTAAATGTCCAAATAAGG	TTGCCCACCACCCTTTTCCAGCCTG
HBZ	AATTGGTGGACGGGCTATTATCC	CGCGGCTTTCCTCTTCTAAGG	ACCGTCAAGCACAGCCTCCTCCTCC
Perforin	AGTGCCGCTTCTACAGTTTC	GGTGCCGTAGTTGGAGATAAG	CACCACTTCAACGCCTCCACCCAGC
Granzyme	TGCATCTTGGTCCGATACTCTG	GGCTTCCAGAATCTCCATTGC	AGTCTCTTCCACCTCGGAGGCTTCCAG
β2 microglobulin	TTGTCTTTCAGCAAGGACTGG	CCACTTAACTATCTTGGGCTGTG	TCACATGGTTCACACGGCAGGCAT

HBZ: HTLV-1 basic leucine zipper factor

**Table 2:** tax/β2 and HBZ/ β2 gene expression in ATL and ACs

	Group	Mean	Minimum	Maximum	SEM	SD	*P-*value
tax	ATL	0.1993	0.000004	2.333	0.16499	0.61737	0.011
	ACs	0.00008	0.000012	0.00023	0.00005	0.00010	
HBZ	ATL	0.10176	0.0023	0.8382	0.06131	0.2294	0.0001
	ACs	0.00007	0.0000004	0.00018	0.00002	0.00007	

ATL: Adult T cell Leukemia; HBZ: HTLV-1 basic leucine zipper factor

1Std. Error of mean

2Std. Deviation

Significant differences in tax and HBZ gene expression were observed between ATL patients and ACs

**Table 3:** HTLV-1 proviral load results in ATL and ACs

Group	Mean	Minimum	Maximum	SEM	SD	*P*-value
ATL	12525.61	53	58983	4121.24	17484.994	0.015
ACs	454.30	21	1218	189.9	465.260	
						

ATL: Adult T cell Leukemia; HBZ: HTLV-1 basic leucine zipper factor

Significant differences in proviral load expression was observed between ATL patients and ACs

**Table 4:** Perforin/ β2 and granzyme/ β2 gene expression in ATL patients and healthy carrier groups

	Group	Mean	Minimum	Maximum	SEM	SD	*P*-value
Perforin	ATL	0.0021	0.000002	0.8382	0.06131	0.2294	0.002
	ACs	0.1137	0.0016	0.9802	0.0748	0.2697	
Granzyme	ATL	0.00001	0.0000001	0.00006	0.000006	0.00002	0.036
	ACs	0.00007	0.0000004	0.00018	0.00002	0.00007	

ATL: Adult T cell Leukemia; HBZ: HTLV-1 basic leucine zipper factor

## Discussion

Anti-viral activity of NKCs and CD8^+^ cytotoxic T lymphocytes is mediated by the recognition and lysis of the infected cells. Cytotoxic activity of NKCs and CTLs play a pivotal role in the protection of the organism against viral infections (12). The major cytotoxic mechanism by which CTLs kill the infected cells is exocytosis of secretory granule components such as perforin and pro-apoptotic serine proteases including granzyme, which synergistically kills target cells by various unscheduled apoptotic pathways (15). The role of NKCs in HTLV-1 infection remains unknown, however, a study by Yu *et al* showed that the percentages of NKCs cell subsets and activity significantly decreased in HAM/TSP patients compared with ACs (20). 

Impairment of CTL-mediated lysis has been well documented in chronic human immunodeficiency virus (HIV), cytomegalovirus (CMV), and Epstein-Barr virus (EBV2) infections, especially when the load of the antigen is high (21). The results of the present study showed that the expression of perforin and granzyme B in ATL patients is significantly lower than those of ACs. Sabouri *et al.* reported that the frequency of perforin and GzmB^+^CD8^+^, CD8^+^CD28^-^and CD8^+^CD27^-^T cells in PBMCs of HAM/TSP and HTLV-1carriers is lower than healthy controls (HCs), and a marginal negative correlation was observed between percent perforin-positive CD8^+^ T cells and HTLV-1 PVL in all HTLV-1infected subjects (including patients with HAM/TSP and ACs). The Efficiency of CTL response which is related to perforin and granzyme content is a reflection of the state of CTL activation that is a result of the balance between CTLs and HTLV-1 antigens (18). Furthermore, another study reports that cytotoxic activity and antibody-dependent cell-mediated cytotoxicity were also lower in NKCs from HAM/TSP patients than those of the control group (22). 

While there is a lack of correlation between CTL frequency and HTLV-1 PVL in HAM/TSP patients, there is a negative correlation between HTLV-1 PVL and CTL mediated cytotoxic potency by which the expression of CTL cytolytic components such as perforin and granzyme is accompanied with lower PVL. Another study demonstrates a positive correlation between Foxp3 and the higher expression levels of perforin and granzyme in HAM/TSP patients compared with ACs and HCs. By infection of Foxp3^+^ T cells, HTLV-1 can evade the immune response by the release of perforin and granzyme from Foxp3 infected T cells. Therefore, the expansion of Foxp3 infected T cells may lead to High PVL in HAM/TSP patients (23, 24). 

The frequency of specific CTLs in the patients with ATL is low and this issue might explain why these patients exhibit immune dysfunction. Anti-Tax CD8^+^ T cells are significantly more abundant in ACs compared with ATL patients strongly suggesting that anti-Tax CD8^+^ T cells are involved in the prevention of the development of ATL (17).

Based on our findings, low levels of CTL lytic contents in ATL patients describe the ineffectiveness of anti-viral immune response in ATL disease. Therefore, the higher expression levels of perforin and granzyme in ACs can control the infection and prevent the development of HTLV-1 related diseases (25).

Our results are consistent with previous studies that showed a lack of cytotoxic molecules in ATL cells and low expression of perforin and granzyme B in anti-HTLV-1 CD8^+^ T cells of patients with ATL, which leads to the increased risks of ATL development (17). Studies have shown that the levels of anti-apoptotic and pro-apoptotic proteins are dysregulated in HTLV-1 infected cells (26). In this issue, tax protein has been implicated in apoptosis resistance of infected cells through up-regulation of anti-apoptotic proteins (27). Furthermore, it has been shown that HBZ plays a critical role in the maintenance of HTLV-1-induced transformation in ATL cell lines (28). 

In the present study, we could not find any correlation between the expression of perforin and granzyme B with PVL, tax, and HBZ, which more likely might suggest that viral factors are not involved in the suppression of these genes and other factors may be involved. Migueles *et al*. have reported that long-term non progression HIV-1 infection is associated with perforin expression and proliferative capacity (29). Decreased perforin and granzyme B expression have been also observed in senescent HIV-1-specific CTLs and it might suggest that CTL “exhaustion” could lead to hypofunction (30). In HTLV infection it has been shown that the number and production rates of NKCs are lower than in young healthy subjects, while this issue is similar to elderly healthy subjects, suggesting production of effective CTL molecules such as perforin and granzyme B is disturbed by chronic HTLV-1 infection (31). 

## Conclusion

According to the findings of our study, it could be concluded that low levels of perforin and granzyme B in ATL patients might be associated with CTLs and NKCs hypofunction which supports disease outcome in patients with ATL.
